# Fabrication of Ultra-Fine-Grained W-TiC Alloys by a Simple Ball-Milling and Hydrogen Reduction Method

**DOI:** 10.3390/ma14195865

**Published:** 2021-10-07

**Authors:** Shaoting Lang, Ningbo Sun, Junhui Cao, Weixin Yu, Zhijun Yang, Shusen Hou

**Affiliations:** 1 School of Mechanical and Electrical Engineering, Xinxiang University, Xinxiang 453000, China; shaotinglang@163.com (S.L.); caojunhui@me.com (J.C.); yuweixin2012@163.com (W.Y.); yzj165@163.com (Z.Y.); shusen_hou@163.com (S.H.); 2 Foshan Institute for New Materials, Foshan 528200, China; 3 School of Chemistry and Materials Engineering, Xinxiang University, Xinxiang 453000, China

**Keywords:** W-TiC alloys, ball-milling method, core-shell structure, mechanical properties, microstructures

## Abstract

In this paper, a simple method to fulfill the ideal microstructural design of particle reinforced tungsten (W) alloys with promising mechanical properties is presented. W-0.5 wt.% TiC powders with core-shell (TiC/W) structure are prepared by ball-milling and controlled hydrogen reduction processes. TEM observation demonstrates that the nano TiC particles are well coated by tungsten. The W-TiC powders are sintered by spark plasma sintering (SPS) under 1600 °C. The sintered microstructures are characterized by FESEM and TEM. It is found that the W-0.5TiC alloys obtain an ultra-fine-sized tungsten grain of approximately 0.7 μm. The TiC particles with the original nano sizes are uniformly distributed both in tungsten grain interiors and at tungsten grain boundaries with a high number density. No large agglomerates of TiC particles are detected in the microstructure. The average diameter of the TiC particles in the tungsten matrix is approximately 47.1 nm. The mechanical tests of W-0.5 TiC alloy show a significantly high microhardness and bending fracture strength of 785 Hv_0.2_ and 1132.7 MPa, respectively, which are higher than the values obtained in previous works. These results indicate that the methods used in our work are very promising to fabricate particle-dispersion-strengthened tungsten-based alloys with high performances.

## 1. Introduction

Tungsten and its alloys have been used for many engineering applications such as in the aerospace, automotive, nuclear energy, materials processing, and defense industries. They are also considered attractive materials for high-temperature applications such as heating elements, rocket nozzles, heat shields, and combustion chambers [1,2,3]. This is due to the superior properties of tungsten such as high melting point (3420 °C), high thermal conductivity, high tensile strength, and high hardness [1,2,3,4,5]. In addition, due to its excellent radiation shielding properties, tungsten is also considered a promising plasma-facing material for future nuclear fusion reactors [5,6,7]. However, as a body-centered cubic (bcc) metal, it is well known that tungsten shows brittle behavior at lower temperatures, especially below the ductile-brittle transition temperature (DBTT) [8,9]. Generally, pure tungsten tends to crack more easily at room temperature than at temperatures higher than 400 °C, which restricts its possible applications as structural materials.

Transition and rare metal carbide and oxide particles such as ZrC [10,11], TiC [12,13,14,15,16,17], HfC [18], La_2_O_3_ [19,20], and Y_2_O_3_ [21] have been introduced into the tungsten matrix to improve the mechanical properties of tungsten materials effectively. Among these introduced particles, transition metal carbide particles have been proven to be ideal strengthening phases for tungsten materials [22]. This is attributed to their high hardness, high melting temperature, excellent high-temperature strength, and good corrosion resistance [22,23]. In addition, it has been reported that the introduced carbide particles can combine with impurity oxygen at the tungsten grain boundary, which can effectively reduce the embrittlement effect of oxygen impurity [10,24]. More importantly, carbide particles can form coherent or semi-coherent interfaces with tungsten grains, which play an important role in strengthening materials [24,25,26]. As a result, the introduced carbide particles in tungsten matrix can inhibit tungsten grain growth and stabilize the microstructure under high-temperature service conditions. In addition, the dispersed carbide particles can increase the mechanical properties and recrystallization temperature of tungsten materials.

To date, several methods have been developed to prepare carbide particle reinforced tungsten materials, such as using mechanical alloying [3,10,13,14,15,21,27] and chemical method [16,17,28,29,30,31,32] to prepare tungsten composite powders, and then utilizing different sintering methods to consolidate the composite powders. Among these powder preparing methods, mechanical alloying is the predominant preparation method to prepare carbide particle reinforced tungsten alloys. However, mechanical alloying suffers from the drawback of agglomeration or precipitation at grain boundaries and is easy to introduce other low melting point phases into tungsten matrix [10,25,33]. In addition, the chemical method always has to use hydrochloric acid (or other strong acids) as reactant, which inevitably results in the formation of ammonium salt, which is potentially dangerous during the high-temperature hydrogen reduction process. Therefore, new methods are highly required to prepare carbide particle-reinforced tungsten materials.

In present work, a simple ball-milling and controlled hydrogen reduction methods were proposed to prepare the W-TiC composite powder with core-shell structure. The amount of TiC added in tungsten was chosen to be 0.5 wt.% to avoid agglomeration of excessive TiC particles during the preparation processes. After that, SPS was carried out on this kind of composite powder to prepare W-TiC alloys. An ideal microstructure for particle dispersed tungsten alloys and high mechanical performances of the sintered W-TiC alloy were obtained in this paper.

## 2. Materials and Methods

**Figure 1:**
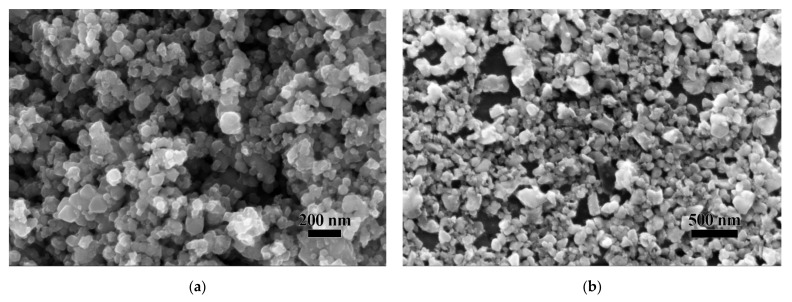
FESEM images of (**a**) the as-obtained nano TiC powders and (**b**) the prepared WO_3_ powders.

The nano TiC powders with an average particle size of 50 nm (see Figure 1a) were purchased from Kaier Nano, Hefei, China. The WO_3_ powders were prepared by calcining of ammonium meta-tungstate (AMT, (NH_4_)_6_H_2_W_12_O_40_•xH_2_O) at 500 °C for 3 h. The as-obtained nano TiC powders and the prepared WO_3_ powders were weighed and added into an agate jar with a weight ratio of 99.5:0.5 (m_W_: m_TiC_). The mixed powders were then subjected to ball-milling in a planetary ball mill for 2 h with a ball-to-powder weight ratio of 10:1 and a rotation speed of 350 rpm.

The reduction process employed a tube furnace (OTF-1200X, Kejing, Hefei, China) under high-purity hydrogen atmosphere. The ball-milled powders were treated at 600 °C for 2 h and at 800 °C for 0.5 h at a heating rate of 5 °C·min^−1^. Then, the powders were cooled down naturally along with the furnace. A spark plasma sintering (SPS) system (Model 1050, Sumitomo Coal Mining Co. Ltd., Japan) was used to densify the reduced powders. The powders were packed into a graphite die and then were subjected to SPS process under vacuum. The samples were sintered at 1600 °C and 50 MPa for a dwell time of 1 min. The size of the as-sintered samples was about 20 mm in diameter and 4.0–5.0 mm in thickness. Four samples were prepared and characterized as follows.

The sintered samples were mechanically ground prior to testing in order to remove the surface contamination during SPS sintering. The densities of the sintered W-TiC samples were measured by the Archimedes principle. The relative densities were calculated from the volume fractions and theoretical densities of tungsten (19.25 g·cm^−3^) and TiC (4.93 g·cm^−3^). The morphologies of the powders and the sintered microstructures were characterized using a transmission electron microscopy (TEM) and a field emission scanning electron microscopy (FESEM, Zeiss Ultra 55, Oberkochen, Germany) equipped with an angle-selective backscattered (AsB) electron detector. A FESEM (Zeiss AURIGA, Oberkochen, Germany) equipped with a focused ion beam (FIB) system was used to prepare the TEM sample of W-TiC alloy and observe the microstructures of the prepared sample. The preparation of TEM sample via FIB cutting technique used a Ga^+^ ions beam and an accelerating voltage of 30 kV. During the final polishing step, a current of 50 pA was used to minimize Ga^+^ ion damage. The crystal structure was identified by X-ray diffraction (XRD, Rigaku Ultima IV, Tokyo, Japan) with a Cu-K_α_ radiation source (λ = 1.5406 nm), which was generated at 40 kV and 40 mA. The scanning angle range was from 10° to 100° at a scanning speed of 10°/min. Vickers micro-hardness tests were performed under a load of 1.96 N for 10 s. The bending strengths were measured by 3-point bending test with a working span of 10 mm at a cross-head speed of 0.5 mm·min^−1^. The dimensions of the specimen were 3 × 2 × 16 mm^3^. Five specimens were tested for each W-TiC sample. The particle size and distribution of the TiC particles were studied by measuring and counting all the particles (more than 300 particles) from the acquired SEM images.

## 3. Results and Discussion

The SEM image of the as-received nano TiC powder is shown in Figure 1a. The average particle size of the nano TiC powder was 50 nm. However, it can be noticed in Figure 1a that the actual particle size of the nano TiC particles was in the range of ~10 to 200 nm, and the particles inclined to aggregate into larger particles. The SEM image of the prepared WO_3_ powder is shown in Figure 1b. The prepared WO_3_ powders showed an irregular shape with particle sizes in the range of 20 to 300 nm.

**Figure 2:**
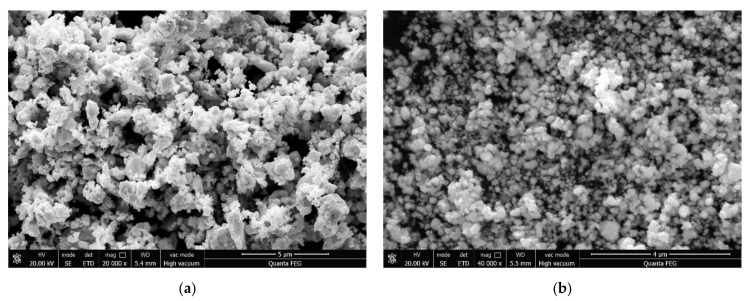
FESEM images of (**a**) the ball-milled WO_3_-TiC powders and (**b**) the reduced W-TiC powders.

The morphologies of the ball-milled WO_3_-TiC powders and the reduced W-TiC powders are shown in Figure 2. It can be noticed that the ball-milled WO_3_-TiC powders showed an aggregated morphology (see in Figure 2a). The original nano powders were milled together and formed larger particles with sizes as large as 2.5 μm. This was due to the reduplicative cold welding and fracturing during the ball-milling process. The reduced W-TiC powders displayed a near-spherical morphology with particle sizes in the range of 20 to 600 nm (see in Figure 2a). In addition, it can be noticed that a fraction of the small particles were huddled together and formed larger-sized aggregates.

**Figure 3:**
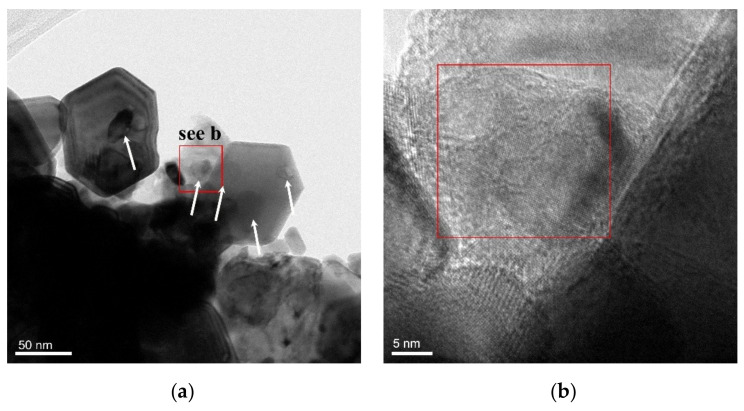

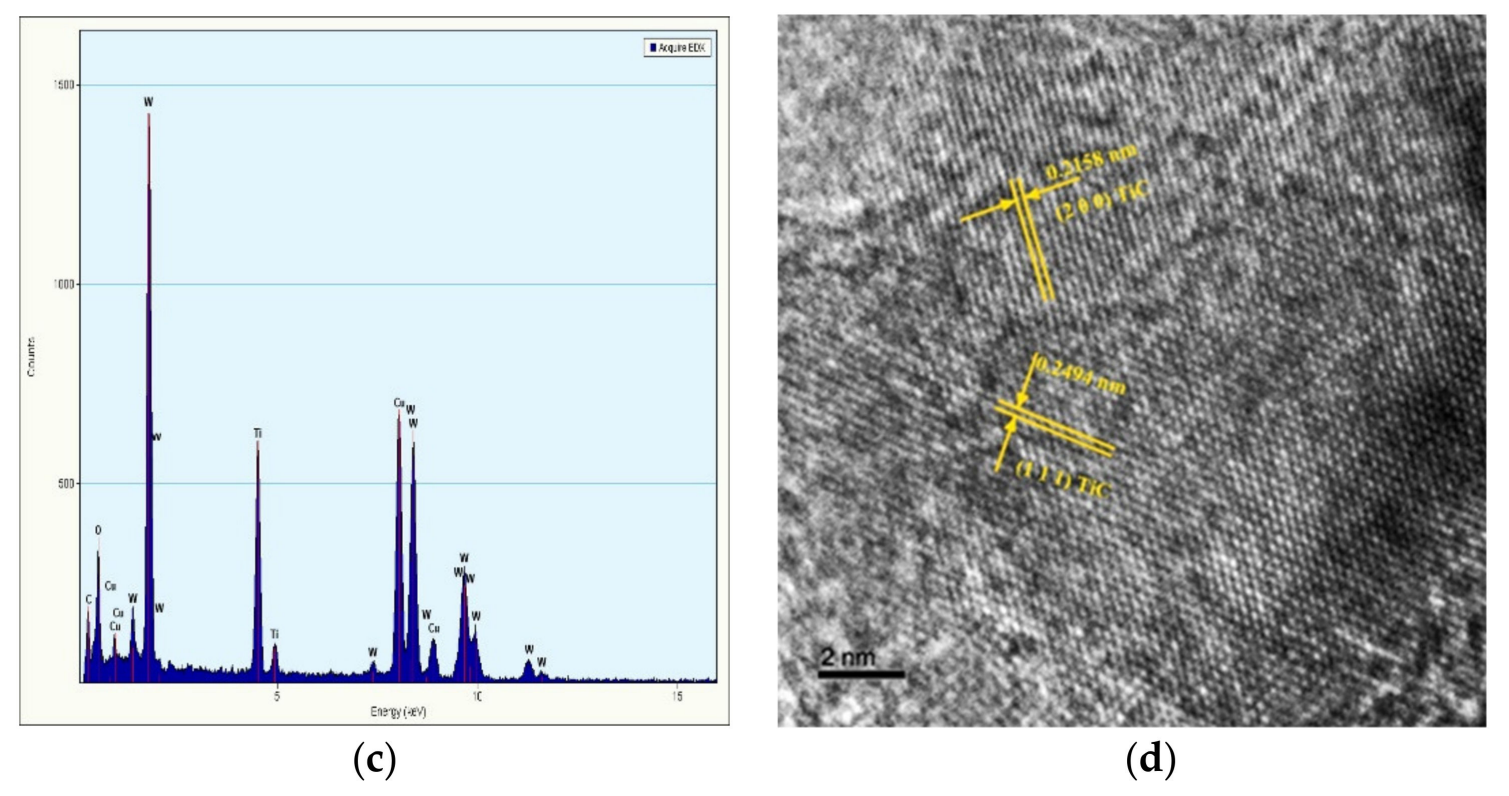
(**a**,**b**) TEM images of the reduced W-TiC powders, (**c**) EDS spectrum of the selected rectangle region in (**b**), and (**d**) high-resolution TEM image of the selected rectangle region in (**b**).

Figure 3a,b show the TEM morphologies of the reduced W-TiC powders. It can be clearly noticed that several small particles with sizes between 10 to 30 nm were perfectly located inside the tungsten particles (as indicated by white arrows in Figure 3a). Figure 3b displayed the high-resolution TEM image of the rectangular area in Figure 3a, which clearly displayed a heterogeneous particle inside a larger particle. EDS spectrum of the selected rectangle region in Figure 3b is shown in Figure 3c. Peaks for the elements of C, O, Ti, W, and Cu were observed in the spectrum. The Cu element originated from the adoption of the copper grid. The O element may come from contamination and oxidation of tungsten. The high-resolution TEM image of the selected rectangle region in b shows two different lattice spacings of 0.2158 and 0.2494 nm, which is consistent with the distance for (2 0 0) and (1 1 1) lattice spacing in face-centered cubic TiC, respectively. Accordingly, these results could reveal that the nano particles inside the tungsten particles were TiC particles.

**Figure 4:**
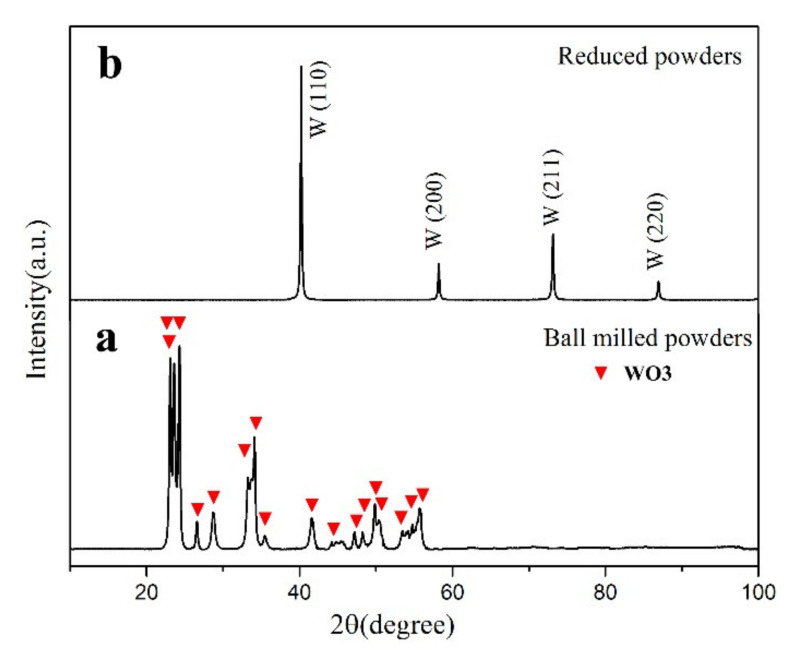
XRD patterns of the (**a**) ball-milled and (**b**) reduced powders.

XRD patterns of the ball-milled and reduced powders are shown in Figure 4. All the peaks of the ball-milled powders were consistent with monoclinic WO_3_ (JCPDS # 71–2141). The peaks of the reduced powders were consistent with standard body-centered cubic tungsten (JCPDS # 04–0806). No TiC phase or other impurities were observed in the XRD patterns of the two kinds of powders. This may be due to that the TiC particles were coated by WO_3_ or tungsten particles.

**Figure 5:**
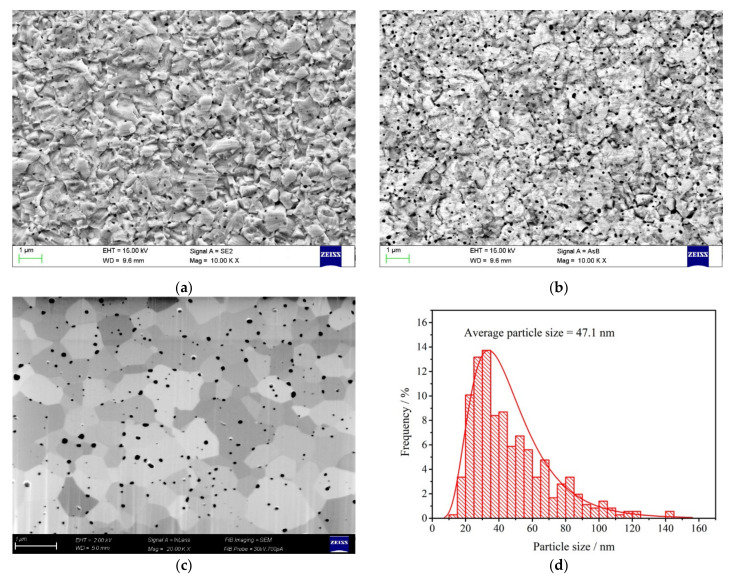
(**a**,**b**) FESEM images of the sintered W-TiC alloys acquired from the SE detector and the AsB detector, (**c**) FESEM images of the sintered W-TiC alloys after FIB polishing, and (**d**) particle size distribution of the TiC particles in (**c**).

FESEM images of the sintered W-TiC alloys acquired from the SE detector and from the AsB detector are shown in Figure 5a,b, respectively. The sintered W-TiC alloy showed an equiaxed grain structure with a number of black TiC particles distributed in the tungsten matrix (see in Figure 5a). Besides, the sintered W-TiC alloy also showed a compact microstructure that no obvious pores were observed at the tungsten grain boundaries, indicating the high density of the sintered sample. FESEM image of the sintered W-TiC alloys acquired from the AsB detector (Figure 5b) was adopted to investigate the distribution of TiC particles in tungsten matrix. It can be noticed from Figure 5b that the black TiC particles with sizes in the range of ~10 to 200 nm were uniformly distributed both at the tungsten grain boundaries and in the tungsten grain interiors. In addition, it should be noted that the sizes of the TiC particles were in accordance with the sizes of the original TiC particles (see Figure 1a), which indicated that the original aggregated TiC particles could be well dispersed and separated by this ball-milling method [34]. This phenomenon can be further verified by the FESEM images of the sintered W-TiC alloys after FIB polishing (Figure 5c). The fine distribution of TiC particles and the tungsten grain (and sub-grain) boundaries can be clearly observed in Figure 5c. The average tungsten grain size was approximately 0.7 μm. The size distribution of TiC particles in Figure 5c is shown in Figure 5d. The average TiC particle size was approximately 47.1 nm, which is almost the same as the original TiC particle size.

**Figure 6:**
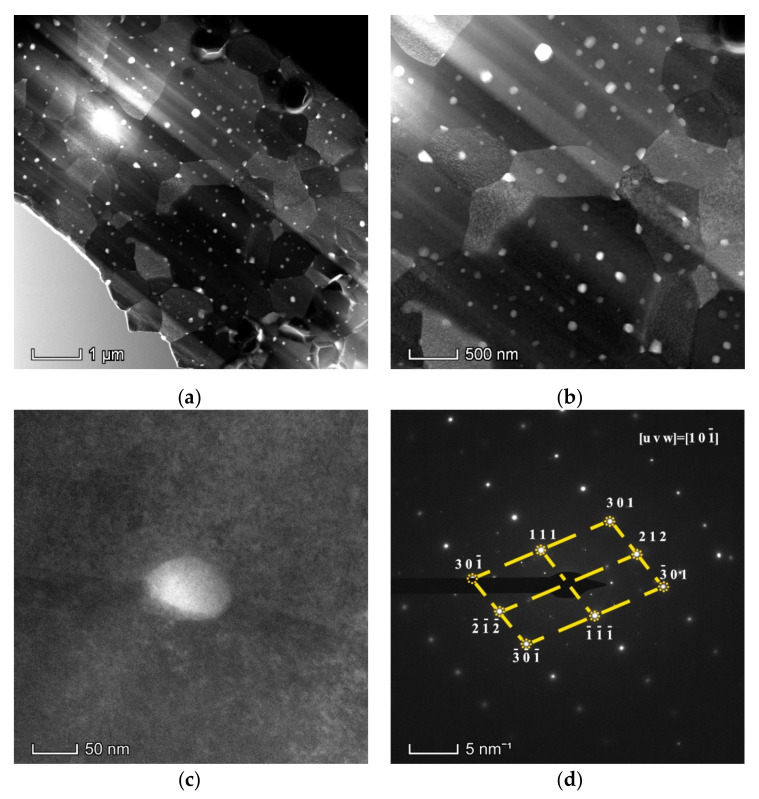
(**a**–**c**) TEM images of the sintered W-TiC alloys, and (**d**) selected area electron diffraction (SAED) image of the particle in (**c**).

**Figure 7:**
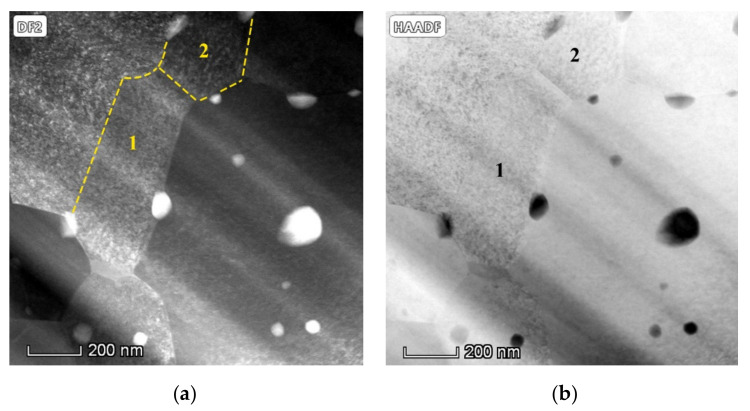
(**a**) Dark-field (DF) and (**b**) high-angle annular dark-field (HAADF) TEM images of the sintered W-TiC alloys, the dotted line in (**a**) marks the sub-grain boundaries, and the numbers 1 and 2 indicate the two sub-grains.

SEM images of the surface microstructure can only provide a two-dimensional view of the distribution of the dispersed TiC particles in tungsten matrix. Hence, simple SEM characterization cannot achieve a full understanding of the spatial distribution of the introduced nano TiC particles in the sintered tungsten matrix. TEM images of the sintered W-TiC alloys were shown in Figure 6a,b, which revealed the distribution of the TiC particles within a certain thickness of the tungsten sample. It can be noticed that the introduced nano TiC particles were uniformly distributed in the tungsten matrix with a high share in the tungsten grain interiors. It should be noticed that the nano TiC particles can be uniformly distributed in each tungsten grain with a high number density and without forming large agglomerates, and each TiC particle can be dispersed separately. This state of particle distribution can significantly inhibit the tungsten grain growth and the Ostwald ripening of TiC particles during high-temperature sintering. This is also the reason for the formation of the ultra-fine-sized tungsten grains. Figure 6c,d show a single particle at the tungsten grain boundaries and the SAED image of the particle, respectively. The measured interplanar spacings and angles between different crystal planes in Figure 6d were in good agreement with the fcc TiC lattice with zone axis of [1 0 −1], which proved that this particle was cubic TiC phase. Figure 7a,b display the dark-field and HAADF TEM images of the sintered W-TiC alloys. Two sub-tungsten grains (grain 1 and 2) were observed in the images, and nano TiC particles can be observed on the sub-tungsten grain boundaries. This proved that the introduced nano TiC particles can also inhibit the sub-tungsten grain growth during sintering.

Table 1 shows the physical and mechanical properties of the sintered W-TiC alloys prepared by different synthesis methods. The relative density was 97.65% for the sintered W-0.5TiC alloys in this work. The microhardness achieved 785 Hv_0.2_ and the bending fracture strength achieved 1132.7 MPa. Compared with the as-sintered W-0.5TiC alloys prepared by wet-chemical method and ball-milling method from the literature [34], the W-0.5TiC alloy in this research showed finer grain sizes and significantly higher mechanical properties. The W-0.5TiC alloy prepared by J. Tan [35] by high-energy ball-milling and SPS obtained an ultra-fine grain size of 0.5 μm and bending strength of 1021 MPa. It was also reported that the bending strength was near 800 MPa for the W-1 wt.% TiC alloy produced by mechanical alloying and hot isostatic pressing (HIP) [36]. Compared with these previous results, our W-TiC alloy prepared by ball-milling and low-temperature hydrogen reduction method presented much higher mechanical properties. These results proved that the synthesis method in this work has large advantages to prepare W-TiC alloys with high performances. However, the W-TiC alloys in this research showed significantly lower bending strength than that of the W-0.5TiC alloy from the literature [13]. The W-0.5TiC alloy prepared via mechanical alloying and HIP achieved a bending strength value of 1600–2000 MPa. This was owing to its much finer grain sizes (0.129 μm) and highly densified microstructure (relative density of ~99%). This also informed us that the mechanical properties of our prepared W-TiC alloys can be further improved by grain refinement and achieve high densification in the future by optimizing the synthesis methods.

**Figure 8:**
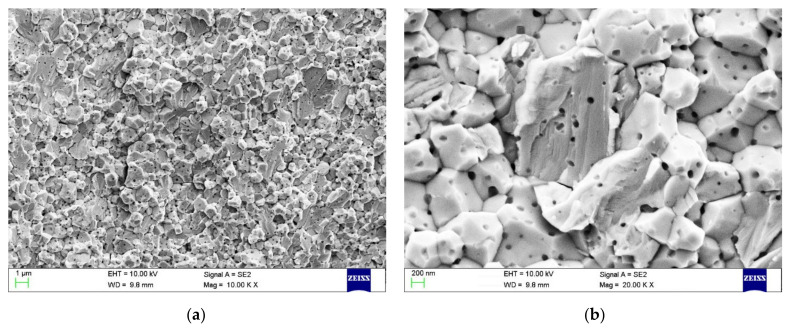
(**a**,**b**) FESEM images of the fractured surfaces of the sintered W-0.5TiC alloys.

The FESEM images of the fractured surfaces of the sintered W-0.5 TiC alloys after 3-point bending test are shown in Figure 8. The fractured surface of the sintered W-0.5 TiC alloys showed both inter-granular fracture and trans-granular fracture morphologies. In addition, TiC particles in the nano-scale range could be detected on every intergranularly fractured grain surface, which verified the excellent dispersing state of the nano TiC particles on the tungsten grain boundaries. Moreover, as shown in Figure 8b, fine dispersed nano TiC particles can also be observed on the trans-granularly fractured tungsten grain facets, which also agreed with the distribution of TiC particles in the tungsten grain interiors obtained from the FESEM results.

It has been studied extensively that the size and distribution state of the introduced carbide or oxide particles have significant influences on the microstructure and mechanical performances of the particle dispersed tungsten alloys. The desired microstructure for particle dispersed tungsten alloys is that the introduced second-phase particles are kept in nano range and uniformly dispersed both in tungsten grain interiors and at tungsten grain boundaries, and simultaneously the tungsten grains are as fine as possible. Firstly, the particles in nano range dispersed within tungsten grains can generate, pin down, and accumulate dislocations within the grains, resulting in the improved mechanical properties, especially tensile properties. Secondly, the particles dispersed at the tungsten grain boundaries can hinder the migration of grain boundaries and inhibit the grain growth under high-temperature conditions, finally resulting in the refined tungsten grains after sintering and the increased recrystallization temperature. It has been reported that the refined tungsten grains can significantly increase the areas of grain boundaries and reduce the concentration of impurity atoms (especially O) at the tungsten grain boundaries [10]. Therefore, the detrimental effect of impurity atoms on tungsten grain boundary can be moderated. Thirdly, the particles located on tungsten grain boundaries can lead to the strengthening of grain boundaries and hence can increase the fracture strength of tungsten materials. Finally, it has been reported that the high-density grain boundary and particle/tungsten interface can act as traps for irradiation-induced defects and hence suppress the formation of voids and deuterium/helium blisters in tungsten alloys during irradiation tests [37,38,39]. Based on these factors, tungsten alloys with the desired microstructures can offer promising mechanical properties such as high hardness, high strength, high recrystallization temperature, high tensile properties, reduced DBTT, et al. It can also be deduced that the significantly high microhardness and bending fracture strength of the prepared W-0.5TiC alloys were mostly originated from the ultra-fine grain sizes and the uniformly dispersed nano TiC particles with high number densities both in tungsten grain interiors and at tungsten grain boundaries. The large-sized specimens of W-TiC alloys with the desired microstructures will be prepared and their high-temperature performances and tensile properties will be analyzed in the future.

## 4. Conclusions

In this paper, a simple method to prepare particle reinforced tungsten alloys was newly proposed. The as-sintered W-0.5 wt.% TiC alloys achieved an ideal combination of particle-dispersed ultra-fine grain structure and high mechanical properties. The preparation route was composed of ball-milling and controlled hydrogen reduction of WO_3_-TiC composite powders and subsequent spark plasma sintering. The prepared W-0.5 wt.% TiC alloys obtained an ultra-fine-sized tungsten grain of approximately 0.7 μm. The TiC particles with the original nano sizes were uniformly distributed both in tungsten grain interiors and at tungsten grain boundaries with a high number density. No large agglomerates of TiC particles were detected in the microstructure, which proved that each TiC particle can be dispersed separately by the preparation method in this paper. The average diameter of the TiC particles in the tungsten matrix was approximately 47.1 nm. The relative density of the sintered W-0.5TiC alloys was 97.65%. The mechanical tests of the sintered W-0.5 TiC alloys showed a significantly high microhardness and bending fracture strength of 785 Hv_0.2_ and 1132.7 MPa, respectively. These results indicated that the methods used in our work are very promising to fabricate particle-dispersion-strengthened tungsten-based alloys with high performances. The thermal fatigue resistance of the prepared W-TiC alloys as a function of temperature will be investigated in our future work.

## Figures and Tables

**Table 1:** Physical and mechanical properties of the sintered W-TiC alloys prepared by different synthesis methods.

Sample	Synthesis Method	Average Grain Size (μm)	Relative Density (%)	Micro Hardness (Hv0.2)	Bending Strength (MPa)
W-0.5TiC (this work)	ball-milling + hydrogen reduction + SPS	0.7	97.65	785	1132.7
W-0.5TiC-wc [34]	wet-chemical method + SPS	0.91	97.61	739.10	1065.72
W-0.5TiC-bm [34]	ball-milling + SPS	0.80	97.37	753.05	705.87
W-0.5TiC [35]	high-energy ball-milling + SPS	0.5	98.6	655.10	1021
W-1.0TiC [36]	mechanical alloying + HIP	~1.0	95.3	714.28	~800
W-0.5TiC [13]	mechanical alloying + HIP	0.129	~99	-	1600–2000

## Data Availability

The data presented in this study are available on request from the corresponding author.
